# Beneficial effect of immunobiotic strains on attenuation of *Salmonella* induced inflammatory response in human intestinal epithelial cells

**DOI:** 10.1371/journal.pone.0229647

**Published:** 2020-03-09

**Authors:** Paulraj Kanmani, Hojun Kim

**Affiliations:** Department of Korean Medicine, Dongguk University, Goyang, Republic of Korea; University of Illinois at Chicago, UNITED STATES

## Abstract

Probiotic bacteria have the ability to modulate host immune responses and have potent therapeutic functional effects against several diseases, including inflammatory diseases. However, beneficial effects of probiotics are strain specific and their interactions with host immune cells to modulate inflammatory response are largely unknown. Intestinal epithelial cells (IECs), which are the first line of defense against invading pathogens, and connects between commensals/probiotics and immune system; therefore, in this study, we used human IECs to assess the probiotic effects of three selected *Lactobacillus* strains *in vitro*. An HT-29 colonic epithelial cell and HT-29/blood mononuclear cells co-culture system were stimulated with *Lactobacillus* followed by *Salmonella* for different hours, after which the mRNA level of cytokines, β-defensin-2 and negative regulators for TLR signaling and protein levels of ZO-1 and IκB-α were analyzed by real-time polymerase chain reaction and western blot analysis. *L*. *brevis* decreased *Salmonella* induced IL-6, IL-8, MCP-1 and IL-1β levels, whereas *L*. *pentosus* suppressed IL-6 and MCP-1 in HT-29 cells. Moreover, *L*. *brevis* was able to increase the mRNA levels of A20, Tollip, SIGIRR and IRAKM, while *L*. *pentosus* reduced the levels of A20, and IRAKM in response to *Salmonella*. In addition, decrease in protein level of TNF-α and increase in mRNA level of IL-10 was observed in *L*. *brevis* and *L*. *pentosus* treated HT-29 cells. *Lactobacillus* strains were differentially modulated ZO-1 and p-IκB-α in HT-29 cells treated with *Salmonella*. Overall, the results of this study indicate that *Lactobacillus* strains attenuate *Salmonella* induced inflammatory responses through beneficial modulation of TLR negative regulators and the NF-κB pathway.

## Introduction

The human intestine is a home to diverse microbial populations that are known to play crucial roles in human health, such as regulation of immune and metabolic homeostasis, digestion of dietary fibers, and protection against pathogenic invaders [1, 2]. *Lactobacilli* are natural inhabitants in the gut of human where they exert potent probiotic effects including competitive exclusion of pathogens and immunomodulatory functions. Beneficial effects related to *Lactobacillus* ingestion have been reported in several *in vitro* and *in vivo* studies [3, 4]. Therefore, *Lactobacillus* species have been exploited as potent probiotics that can alleviate different ailments associated with the human gut [5]. Probiotics are specific live bacteria capable of improving protective effects of intestinal immune responses against harmful physiological conditions and ameliorating inflammatory responses induced by intestinal pathogens [6]. Probiotic *L*. *brevis* G-101 (isolated from Korean kimchi) and *L*. *brevis* 23017 ameliorates chemical induced colitis [7], as well as mercury induced intestinal damage [8] in mice through the inhibition of MAPKs and NF-κB signaling pathways. Similarly, the strain *L*. *curvatus* WiKim38 was isolated from Korean kimchi that administration has been shown to alleviate dextran sodium sulfate (DSS) induced colitis in mice and to increase the expression of IL-10 in bone marrow-derived DCs *in vitro* by activating NF-κB and ERK pathways [9]. Another study evaluated the prophylactic effect of *L*. *pentosus* S-PT84 against *Candida* infection and gastric inflammation in murine model [10]. In which, they also found to reported that *L*. *pentosus* treatment was able to reduce the severity of lesion in stomach and to prevent adhesion of *Candida* on the stomach. In addition, many clinical and experimental studies have also reported that probiotic *Lactobacillus* ameliorated pathogens as well as traveler’s and antibiotic associated diarrhea [11]. Orally administered probiotic *L*. *casei* were found to survive in the intestinal tract of mice [12], and to be able to reduce the occurrence of acute and incidence of diarrhea in children [13, 14].

*Salmonella* is an intestinal pathogen that causes fever, vomiting and diarrhea, resulting in high morbidity and mortality of people, especially young children. Indeed, approximately 200,000 deaths from salmonellosis are reported annually [15]; therefore, it is considered to be a major public health issue worldwide. As a powerful enteric pathogen, *Salmonella* reach the gut lumen via ingestion of contaminated food, where they induce intestinal inflammation via several pathways, especially activation of Toll-like receptors (TLRs) signaling [15]. Toll-like receptors are the kind of pattern recognition receptors (PRRs) that can be expressed by intestinal epithelial cells (IECs) to respond invading pathogens, including *Salmonella* [16]. Receptors in the membrane have been shown to sense *Salmonella* associated molecular patterns (lipoproteins, LPS, flagellin and CsgA) and mediate signaling cascades, resulting in activation of the nuclear factors κB (NF-κB) pathway, which increases the production of inflammatory cytokines/chemokines [17]. Moreover, TLRs deficient mice were found to be highly sensitive to *Salmonella* infection and to have lower innate immune functions [17].

Uses of probiotic strains have been shown to reduce *Salmonella* infection, as well as its associated diarrheal complications and intestinal inflammation. Probiotic *Lactobacillus* strains were able to prevent *Salmonella* or its lipopolysaccharide (LPS) induced interleukin-8 (IL-8) production and intestinal barrier dysfunction *in vitro* [18, 19]. Another study showed that probiotic *Lactobacillus* efficiently reduced *Salmonella* infection and gut inflammation by increasing the levels of propionic acid and mucin-2, as well as changing the level of tumor necrosis factor-alpha (TNF-a), interleukin-10 (IL-10) and myeloperoxidase (MPO) in mice [20]. Several mechanisms have been proposed for the beneficial activity of probiotics strains; however, they mainly acted through the modulation of TLR signaling. The cell free supernatant (CFS) of *Bifidobacterium breve* dampens *Salmonella* induced pro-inflammatory cytokines/chemokines in human dendritic cells (DCs) via activation of the TLR signaling pathway [21]. In addition, *L*. *paracasei* and its CFS modulate *Salmonella* induced inflammatory response in Caco2/DCs cells through TLR activation [22]. These studies confirmed that probiotics had the ability to reduce pathogenesis of *Salmonella in vitro* and *in vivo*; however, they did not provide detailed descriptions of the molecular mechanisms for the beneficial activity of probiotic strains. In addition, each probiotic strain has different functional properties; therefore, the screening or selection of active probiotic strains with preeminent functional effects is very important to achieving target therapeutic effects. Intestinal epithelial cells (IECs) are the layer of cells lined up on the luminal surface of the intestinal epithelium, play vital roles in the maintenance of gut homeostasis and provide protection against microbial infection [23, 24]. IECs can respond to pathogenic invaders and their associated molecular patterns to reinforce the gut barrier function and improve the innate immunity via TLRs [24]. Probiotic *Lactobacillus* were able to up-regulate TLRs expression and cytokine/chemokine production in IECs and DCs [25, 26]. Many studies have suggested that IECs are a useful *in vitro* model for selection of active probiotic strains and to decipher functional properties of probiotic strains *in vitro*. Therefore, in this study, heterogeneous human colon (HT-29) cells were used to select active probiotic strains with potent immunoregulatory functions against *Salmonella typhimurium* and to study the molecular mechanisms involved in the immunoregulatory activity of probiotic bacteria.

## Materials and methods

### Bacterial strains and cell culture

*Lactobacillus brevis*, *L*. *curvatus* and *L*. *pentosus* were isolated from Korean fermented foods that were characterized phenotypically and genotypically. A single colony of each strain was cultured in deMan-Rogosa-Sharp (MRS) by incubating at 3700B0030C for 19 h. The cultured cells were then centrifuged and washed in distilled phosphate buffer saline (DPBS), suspended in Roswell Park Memorial Institute 1640 medium (RPMI 1640, WELGENE Fresh Media, Gyeongsagbuk-do, South Korea) at appropriate concentrations and stored at -4°C for further cell stimulation. The cytotoxicity of the isolated strains was analyzed cell viability, proliferation and cytotoxicity assay kit (EZ-CYTOX, DOGEN Bio co. Ltd) in HT-29 cells. The enteric pathogen *Salmonella typhimurium* was cultured in Luria bertani (LB) broth for 19 h at 37°C, harvested by centrifugation, washed with DPBS and stored at -4°C by mixing with RPMI media.

The human colon cell (HT-29 and Caco2) lines were procured from the Korean cell line bank (Seoul, South Korea) and maintained in RPMI and DMEM. HT-29 cells were cultured in RPMI medium (Gibco™), while Caco2 cells were maintained in DMEM supplemented with 10% fetal bovine serum (FBS) and 1% penicillin/streptomycin at 37°C under 5% CO_2_. The medium was changed every alternative day for 5–6 days.

Peripheral blood mononuclear cells (PBMCs) were isolated from heparinized blood of healthy volunteers using Histopaque1077 (Sigma). Approved by the institutional review board of Dongguk University. Freshly prepared PBMCs were stored in RPMI medium supplemented with 10% FBS for the co-culture study.

### Anti-inflammatory activity of LABs on HT-29 *in vitro*

To study the potential effects of LABs on modulation of inflammatory cytokine production, *in vitro* experiments were designed with HT-29 cells that were cultured (3 × 10^4^ cells/ml) in 12 well type I collagen coated plates (SPL Life Sciences Co. Ltd., Gyeonggi-do, Korea) at 37°C under 5% CO_2_ for 5–6 days. Confluent monolayers of HT-29 cells were pre-stimulated with *L*. *brevis*, *L*. *curvatus* and *L*. *pentosus* (5 × 10^7^ cells/ml) for 48 h, after which the cells were washed with RPMI medium and post-stimulated with *Salmonella* for 3 and 12 h. Total RNA from treated cells was used to analyze the expression of inflammatory cytokines/chemokine (IL-6, IL-8, MCP-1 and IL-1-β) using RT-PCR as mentioned below.

### Effect of LABs on modulation of TNF-α induced β-defensin-2 expression *in vitro*

Caco2 cells (3 × 10^4^ cells/ml) were cultured in 12 well type I collagen coated plates (SPL Life Sciences Co. Ltd., Gyeonggi-do, Korea) at 37°C under 5% CO_2_. After 15–16 days, different LABs (5 × 10^7^ cells/ml) were added to the confluent cells and incubated at 37°C under 5% CO_2_ for 48 h, then post-stimulated with TNF-α (10 ng/ml) for 12 h. Finally, total RNA from treated cells was extracted and used to analyze the expression of β-defensin-2 by RT-PCR.

### Real-time polymerase chain reaction (RT-PCR)

Total RNA from stimulated cells was extracted by adding TRIzol reagent (Invitrogen). The purity of the RNA was checked, after which it was used to synthesize cDNA using a BioRad thermal cycler (BIORAD, Hercules, CA, USA). Next, RT-PCR was performed using a 7300 real-time PCR system (Roche Applied Science, Indianapolis, IN, USA) with SYBR green and primers [27]. The reaction mixture had a total volume of 20 μl that contained 1 μl of cDNA and 19 μl of master mix including SYBR green and both forward and reverse primers (1 pmol/μl). The amplification was conducted by subjecting the samples to 50°C for 5 min followed by 95°C for 5 min and then 40 cycles of 95°C for 15s, 60–63°C for 30s and 72°C for 30s. β-actin was used as an internal control.

### Effect of LABs on modulation of TLR negative regulators on HT-29 *in vitro*

To analyze the expression of negative regulators of TLRs, HT-29 cells (3 × 10^4^ cells/ml) were cultured in 12 well type I collagen coated plates (SPL Life Sciences Co. Ltd., Gyeonggi-do, Korea) at 37°C under 5% CO_2_ for 5–6 days. The monolayers of cells were then stimulated with *L*. *brevis*, *L*. *curvatus* or *L*. *pentosus* (5 × 10^7^ cells/ml) for 48 h at 37°C under 5% CO_2_, and followed by stimulated with *Salmonella* for 3 and 12 h. Total RNA from the treated cells was then subjected to RT-PCR to analyze the expression of TLR negative regulators (A20, SIGIRR, Tollip, and IRAK-M).

### Co-culture

HT-29 cells were seeded (3.5 × 10^4^ cells/well) on transwell culture inserts [(Transparent PTFE membrane coated collagen, 0.4 μm pore size) Transwell-COL, Corning Incorporated, NY, USA], and cultured at 37°C under 5% CO_2_ for 5–6 days until confluence was reached (TEER value ~601 Ω cm^2^). This setup was put in 6 well cell culture plates for co-culture with PBMCs that were seeded in the lower basolateral chamber. To analyze the anti-inflammatory activity of LABs in the co-culture setup, apical monolayers of HT-29 cells were stimulated with *L*. *brevis*, *L*. *curvatus* and *L*. *pentosus* (5 × 10^7^ cells/ml) for 48 h, then with *Salmonella* for 12 h. The CFS were collected from both apical and basolateral sides and stored at -4°C until estimation of the protein level of TNF-α using a commercially available enzyme-linked immunosorbent assay kit (Human TNF-α Quantikine ELISA kit, R & D system, MN, USA) according to the manufacturer’s instructions. In addition, the expression of IL-10 and TFG-β in HT-29 cells was analyzed by RT-PCR.

### Western blot

To analyze the level of Tight-Junction protein and NF-κB protein, HT-29 cells were cultured (1.8 × 10^5^ cells/dish) in dishes (60 mm) at 37°C under 5% CO_2_ for 5–6 days. Next, HT-29 cells were stimulated with *Salmonella* (2 h), LABs alone (48 h), LABs + *Salmonella* (2 h, *Salmonella* combined treatment), LABs + *Salmonella* (2 h, *Salmonella* post-treatment separately), and or LABs + *Salmonella* (48 h both combined). The treated cells were then washed three times with distilled PBS, after which samples were collected by adding 200 μl of CellLytic M cell lysis reagent (Sigma-Aldrich, St. Louis, MO, USA) containing phosphatase and protease inhibitors. The lysed cells were then scraped and transferred to fresh Eppendorf tubes (1.5 ml), after which they were sonicated at 50% for 3 to 5 sec and stored at -70°C for blotting analysis. The total protein for each sample was estimated using the colorimetric method [(Bicinchoninic acid (BCA) assay kit (Thermo Scientific, Pierce, Rockford, IL)].

For blotting analysis, lysed samples were loaded into 10% SDS-polyacrylamide gels, then electrophoresed to separate the proteins and transferred to a nitrocellulose membrane (Trans-Blot Turbo™, BioRad). The transferred membranes were then cut at the desired part and incubated with blocking buffer for 1–2 h, after which they were incubated with specific primary antibodies of the targeting proteins including the tight junction protein (Zonula occludens-1) and nuclear factor kappa B (p-IκB-α) (Cell Signaling Technology, Beverly, MA, USA), as well as β-actin antibody (Santa Cruz Biotechnology, Inc., Dallas, TX, USA) at 1000 times dilution of their original concentration. The overnight-incubated (4°C) membranes were subsequently washed with TBS-T buffer and incubated with secondary antibody (goat anti-rabbit or anti-mouse IgG-HRP polyclonal antibodies (AbFrontier, Seoul, Korea). After 1–2 h of incubation at 4°C, the membranes were washed with TBS-T and spread with western blot detection solution (Dyne ECL Star, Korea). Finally, the optical protein bands were detected and the densitogram peaks were estimated using the Image J software.

### Statistical analysis

Statistical analyses were performed using the SPSS software package (SPSS 12.0, SPSS Inc., Chicago, IL, USA). Kruskal-Wallis test and pairwise comparison were performed to analyze the significant between groups, and a significant difference was accepted at p<0.05 level.

## Results

### Lactobacillus treatment reduces inflammatory cytokine expression *in vitro*

**Fig 1:**
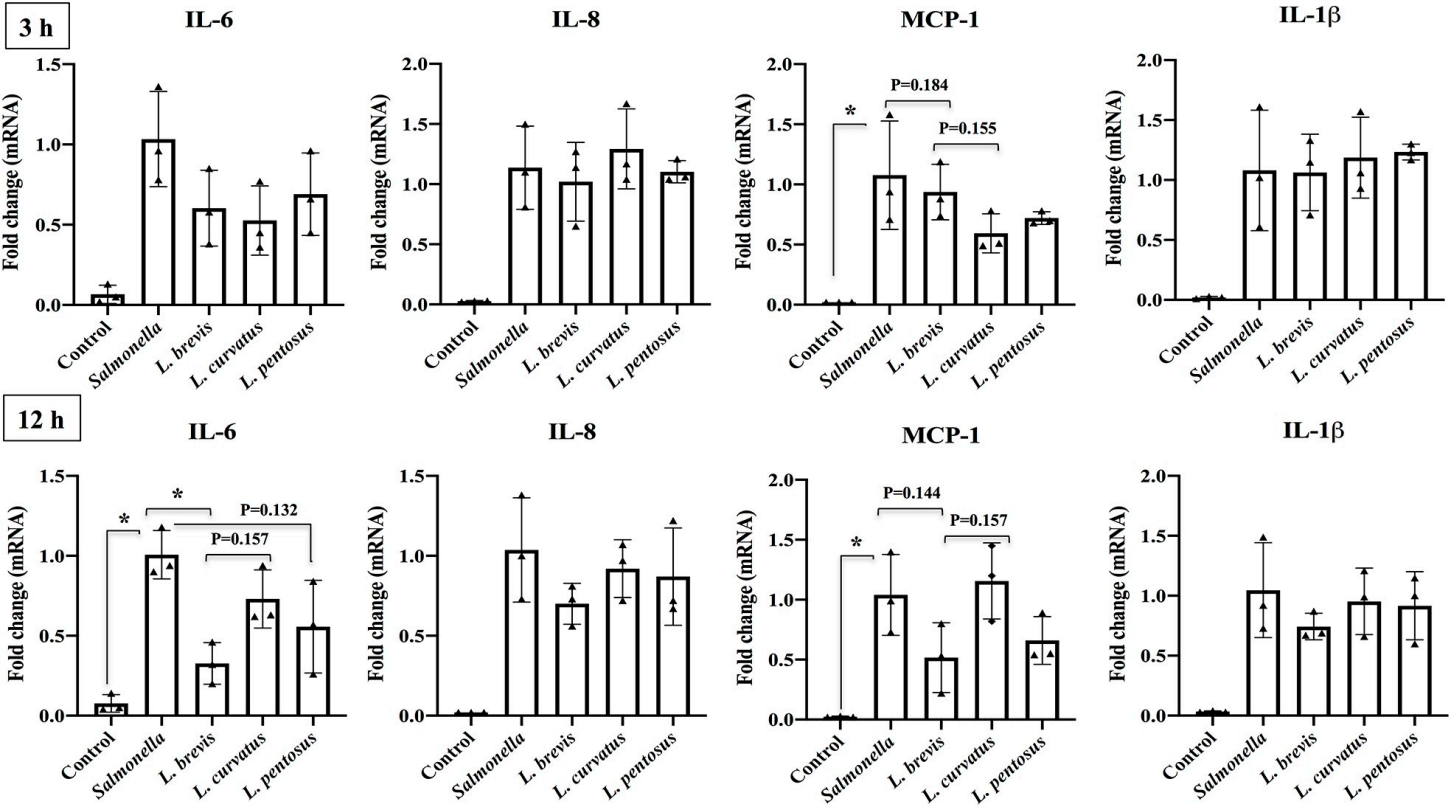
*Lactobacillus* strains reduce *Salmonella* induced cytokines expression in IECs. HT-29 cells were pre-stimulated with *Lactobacillus* strains (5 × 10^7^ cells/ml) for 48 h and then post-stimulation with *Salmonella* for 3 and 12 h. The mRNA level of IL-6, IL-8, MCP-1 and IL-1β were determined by RT-PCR. HT-29 cells either with medium or *Salmonella* alone were used as controls. The results represented here is an average values (mean ± S.D) of three independent values. Significant differences between the groups, *p<0.05.

To analyze the anti-inflammatory activity of different LABs, HT-29 cells were treated with *L*. *brevis*, *L*. *curvatus* and *L*. *pentosus* for 48 h, and then post-stimulated with *Salmonella* for 3 and 12 h. The expression of pro-inflammatory cytokines was subsequently analyzed by RT-PCR. As shown in Fig 1, HT-29 cells stimulated with *Salmonella* increased the expression of IL-6, IL-8, MCP-1 and IL-1β at all stimulation hours. However, these *Salmonella* induced inflammatory responses were attenuated by LABs treatment in a time dependent manner. Stimulation of HT-29 cells with *L*. *brevis*, *L*. *curvatus* and *L*. *pentosus* altered the mRNA level of IL-6 and MCP-1 at 3 h, whereas IL-8 and IL-1β were not altered by LABs in HT-29 cells under inflammatory conditions. Conversely, at 12 h pre-stimulation, LABs revealed different inhibitory patterns in HT-29 cells stimulated with *Salmonella*. All LABs showed reduced levels of IL-6, but the level of MCP-1 was only diminished by *L*. *brevis* and *L*. *pentosus*. Moreover, HT-29 cells stimulated with *L*. *brevis* reduced IL-8 and IL-1β levels, but the reductions were not significant as compared with *Salmonella* in HT-29 cells.

### Lactobacillus affects TNF-α induced β-defensin-2 expression *in vitro*

**Fig 2:**
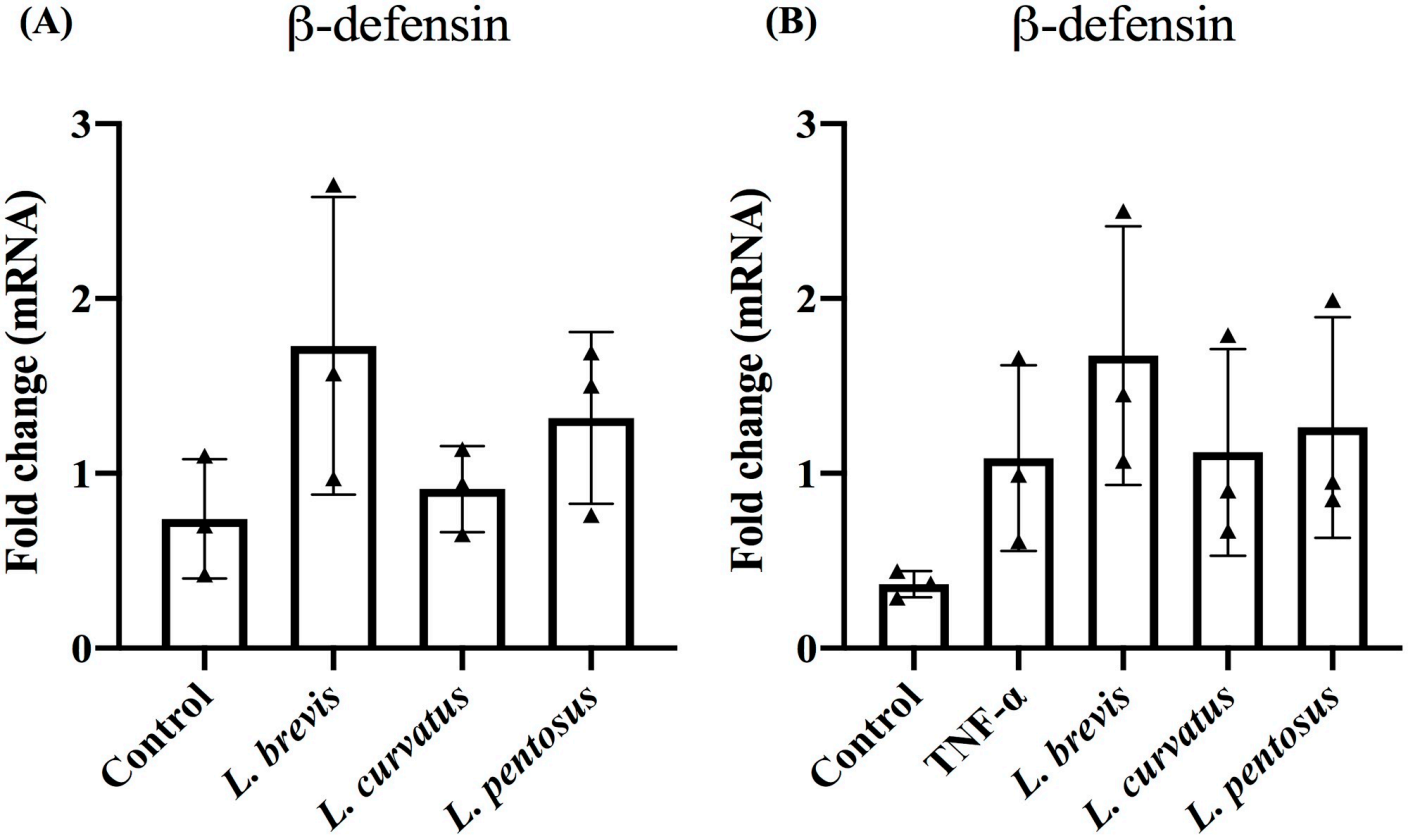
*Lactobacillus* strains modulate TNF-α induced β-defensin-2 expression in IECs. Caco2 cells were pre-stimulated separately with *Lactobacillus* alone (A) and post-stimulation with TNF-α 12 h (B). The expression of β-defensin-2 expression was analyzed by RT-PCR. Caco2 cells with or without presence of TNF-α were used as controls. The results represented here is an average values (mean ± S.D) of three independent values. Significant differences between the groups, *p<0.05.

We next analyzed whether LABs augment β-defensin-2 expression in Caco2 cells or not. To accomplish this, Caco2 cells were incubated with different LABs prior to incubation with TNF-α for 12 h. The expression of β-defensin-2 in Caco2 cells was then analyzed by RT-PCR. Cells treated with LAB alone tended to increase the expression of β-defensin-2, but the increase were not significantly higher than the control (Fig 2A). In addition, TNF-α treatment increased the mRNA level of β-defensin-2, and this was further increased when cells pre-stimulated with *Lactobacillus*. However, *L*. *brevis* showed higher-level of β-defensin-2 than TNF-α, while *L*. *curvatus* and *L*. *pentosus* also increased, but not significantly as compared to TNF-α in Caco2 cells (Fig 2B).

### Lactobacillus modulates negative regulators of TLR signaling in HT-29 cells

**Fig 3:**
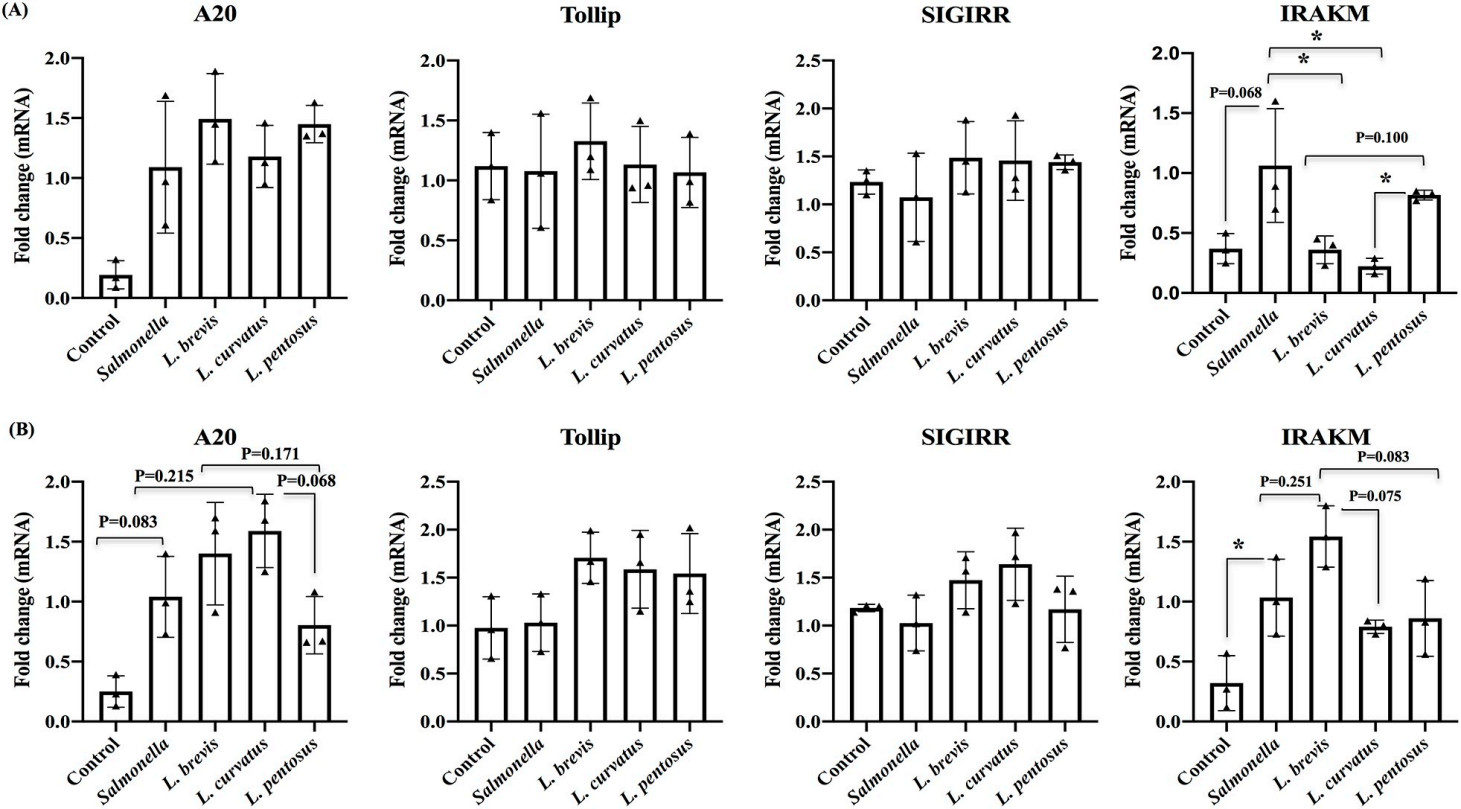
*Lactobacillus* strains modulate TLRs negative regulators expression in HT-29 cells. HT-29 cells were pre-stimulated with *Lactobacillus* strains (5 × 10^7^ cells/ml) for 48 h and then post-stimulation with *Salmonella* for 3 (A) and 12 h (B). The mRNA level of A20, Tollip, SIGIRR and IRAK-M were analyzed by RT-PCR. HT-29 cells either with medium or *Salmonella* alone were used as controls. The results represented here is an average values (mean ± S.D) of three independent values. Significant differences between the groups, *p<0.05.

TLRs are known to play important roles in the activation of host innate and adaptive immunity via binding with microbe associated molecular patterns (MAMPs). However, overactivation of TLRs leads to development of several diseases, including inflammatory diseases, by disturbing immune homeostasis. Accordingly, several molecules negatively control or regulate TLRs signaling pathways *in vitro* or *in vivo*; therefore, we sought to examine whether LABs treatment modulates the expression of TLR negative regulators in HT-29 cells. To accomplish this, HT-29 cells were treated with *L*. *brevis*, *L*. *curvatus* and *L*. *pentosus* for 48 h, then post-stimulated with *Salmonella* for 3 and 12 h. Results of RT-PCR showed that *Salmonella* alone increased the expression of A20 and IRAKM, while the levels of Tollip and SIGIRR were marginal in HT-29 cells at both stimulation hours (Fig 3A and 3B). However, these *Salmonella* induced TLRs negative regulators were modulated by LABs in a time dependent manner. At 3 h, *L*. *brevis* pre-treatment slightly increased the mRNA levels of A20, Tollip and SIGIRR, but these increases were not significantly higher than *Salmonella* (Fig 3A). All LABs except *L*. *pentosus* significantly reduced the level of IRAKM in HT-29 cells. Conversely, HT-29 cells pre-treated with all LABs except *L*. *pentosus* augmented A20, Tollip and SIGIRR levels, and these increases were not significantly higher than the levels induced by *Salmonella* alone (Fig 3B). Furthermore, IRAKM was increased by *L*. *brevis* and it was also not significantly higher than *Salmonella* and other LAB strains.

### Lactobacillus improves tight-junctions between cells in vitro

**Fig 4:**
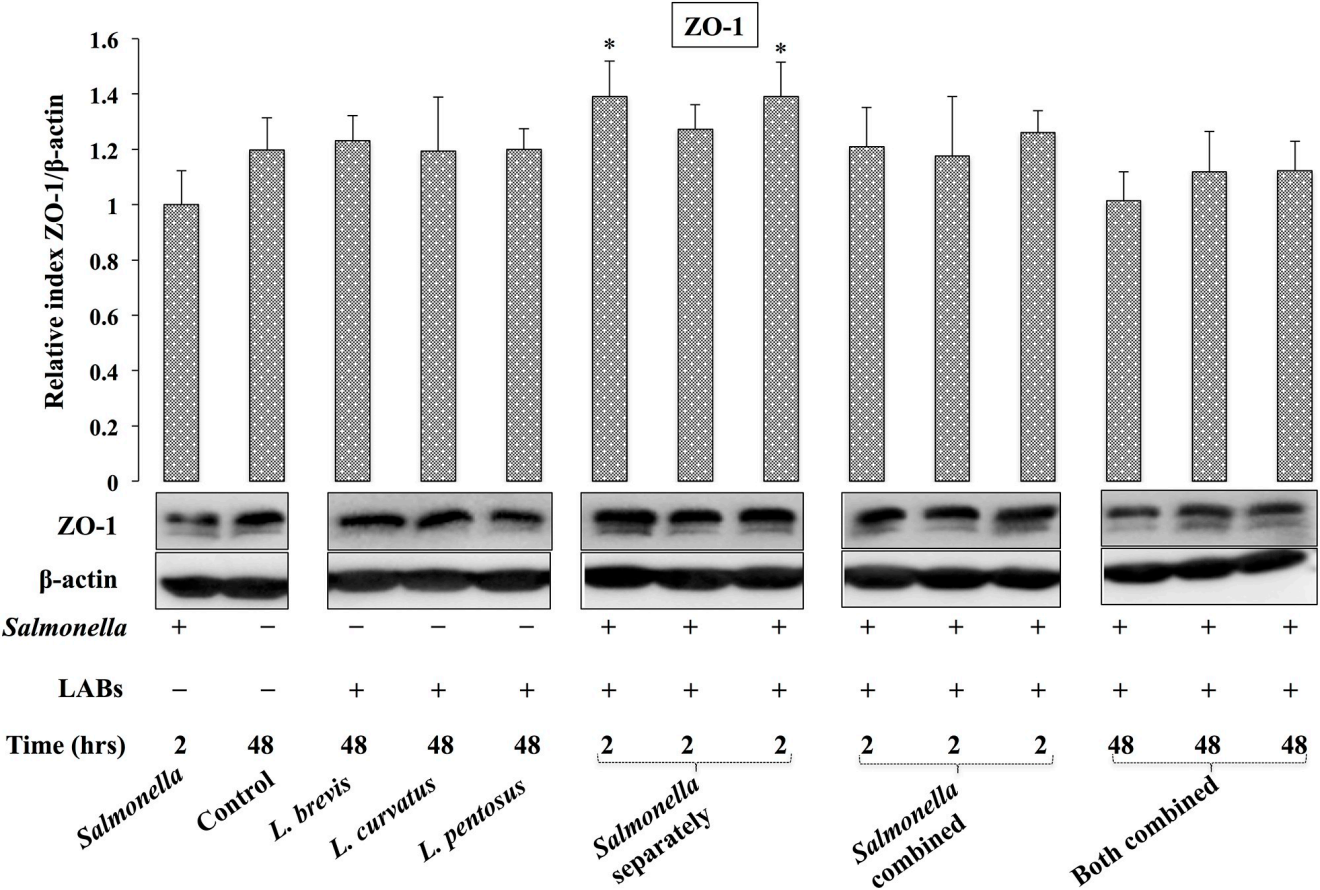
*Lactobacillus* strains ability to modulate tight-junction protein expression in IECs. HT-29 cells were stimulated as follows: *Salmonella* alone, medium alone (control), LABs alone, LABs + *Salmonella* (2 h separately), LABs + *Salmonella* (2 h combined), and LABs + *Salmonella* (48 h). The protein level of Zonula occludens-1 (ZO-1) was analyzed by western blot. The image J software used to determine intensities of proteins bands. Significant differences between the groups, *p<0.05.

We next analyzed whether LABs treatment increases ZO-1 production in HT-29 cells. Briefly, cells were stimulated as described in the methods section and the production of ZO-1 protein was analyzed by western blot. Stimulation of cells with *Salmonella* alone decreased the production of ZO-1, while treatment with LABs alone or with LABs/*Salmonella* (*Salmonella* post-combined stimulation for 2 h) up-regulated the level of ZO-1 in HT-29 cells; however, these increases were relatively similar to those observed in the control (Fig 4). *L*. *brevis* and *L*. *pentosus* pre-treatment for 48 h significantly increased the production of ZO-1 in HT-29 cells that were post-stimulated with *Salmonella* for 2 h. Furthermore, cells treated with LABs + *Salmonella* for 48 h showed marginal level of the ZO-1 that were relatively similar to the levels of control and LABs alone.

### Lactobacillus modulates cytokines production in *in vitro* co-culture system

**Fig 5:**
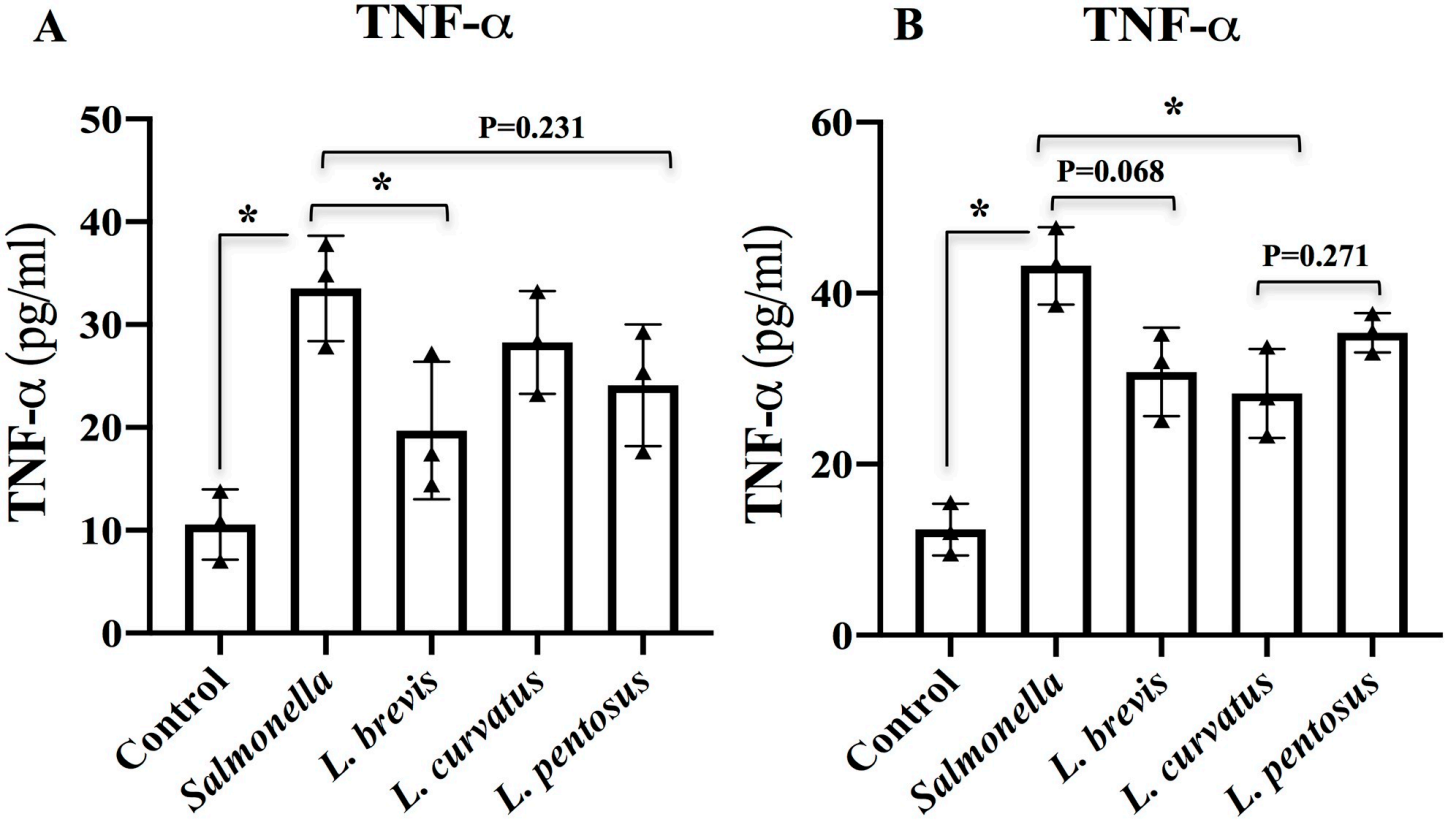
*Lactobacillus* strains ability to reduce TNF-α expression in HT-29 cells co-cultured with PBMCs. Apical HT-29 cells were stimulated with LABs for 48h, followed by stimulated with *Salmonella* for 12 h. The production of TNF-α in the apical (A) and basolateral (B) compartments was determined using ELISA. Cells treated either with medium or *Salmonella* alone were used as controls. The results represented here is an average values (mean ± S.D) of three independent values. Significant differences between the groups, *p<0.05.

We next studied the effect of LABs on modulation of inflammatory and immunoregulatory cytokines production at the protein and mRNA level in co-cultures of PBMCs and HT-29 cells. For this study, apical monolayers of HT-29 cells were treated with LABs for 48 h, and then post-stimulated with *Salmonella* for 12 h. The production of TNF-α at the protein level was quantified using ELISA. We found that *Salmonella* was able to increase the level of TNF-α in both apical (Fig 5A) and basal side (Fig 5B), but these increases were significantly diminished when HT-29 cells were post-stimulated with *L*. *brevis* and *L*. *curvatus*.

**Fig 6:**
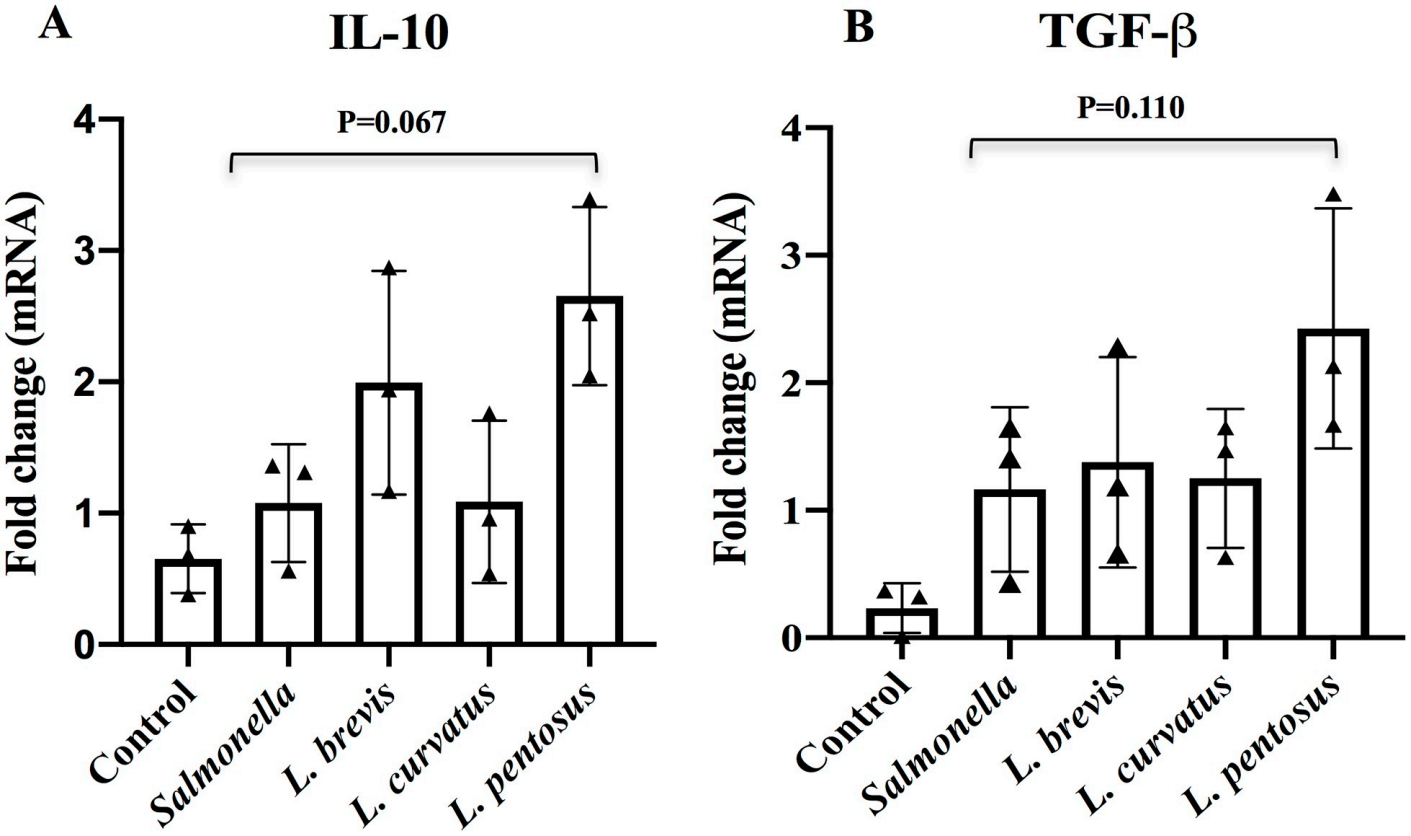
*Lactobacillus* strains ability to increase regulatory cytokines expression in HT-29 cells co-cultured with PBMCs. Apical HT-29 cells were stimulated with LABs for 48h, followed by stimulated with *Salmonella* for 12 h. The expression of IL-10 (A) and TGF-β (B) were analyzed by RT-PCR. Cells treated either with medium or *Salmonella* alone were used as controls. The results represented here is an average values (mean ± S.D) of three independent values. Significant differences between the groups, *p<0.05.

Conversely, all LABs except *L*. *curvatus* tended to increase the mRNA level of IL-10, but these increases were not significantly higher than those observed in the control and *Salmonella* treatments (Fig 6A). In addition, the level of TGF-β was increased by *L*. *penstosus* pre-stimulation, while other LAB strains had no significant effect on the level of TGF-β, which remained relatively similar to those of *Salmonella* treated cells (Fig 6B).

### Lactobacillus modulates the NF-κB pathway *in vitro*

**Fig 7:**
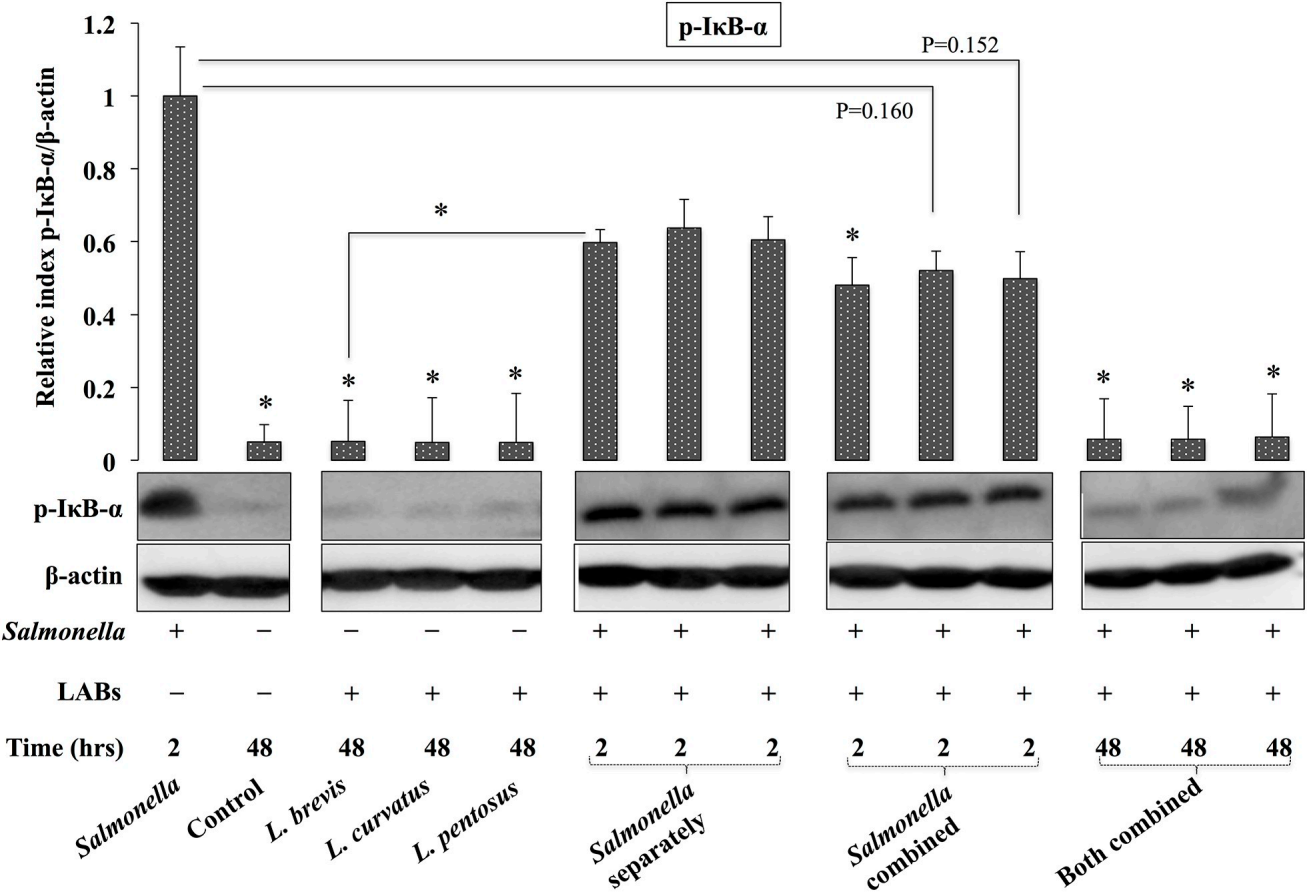
LABs inhibited *Salmonella* induced NF-κB activation *in vitro*. HT-29 cells were stimulated as follows: *Salmonella* alone, medium alone (control), LABs alone, LABs + *Salmonella* (2 h separately), LABs + *Salmonella* (2 h combined), and LABs + *Salmonella* (48 h). The protein level of p-IκB-α was analyzed by western blot. The image J software used to determine intensities of proteins bands. Significant differences between the groups, *p<0.05.

As previously mentioned, *Salmonella* treatment was able to increase the expression of different inflammatory cytokines in HT-29 cells. Indeed, most pathogens induce inflammatory cytokines through activation of the NF-κB pathway *in vitro* or *in vivo*; therefore, we next analyzed whether LABs modulate *Salmonella* induced NF-κB activation in HT-29 cells. The experiments were performed as described in the methods section. Activation of the NF-κB pathway was analyzed based on the IκB-α phosphorylation level in HT-29 cells using western blot analysis. *Salmonella* treatment alone increased the phosphorylation of IκB-α in HT-29 cells, and this was significantly down-regulated following pre-exposure of cells to LAB strains (Fig 7). Treatment with LABs alone (48 h) and co-treatment with *Salmonella* (both combined for 48 h) did not increase the phosphorylation level of IκB-α relative to the control. In addition, as compared to *Salmonella* alone, all three LABs tended to decrease the IκB-α levels in HT-29 cells that were post-treated with *Salmonella* for 2 h and co-treated with *Salmonella* for 2 h and 48 h, suggesting that LAB strains exhibit immunoregulatory effects through the modulation of NF-κB pathway.

## Discussion

Intestinal ECs (IECs) are the dominant cell types in the guts of humans and animals that are continually exposed to gut microbes and mediate signaling to maintain host innate immunity by sensing gut bacteria, including *Lactobacillus*, and their molecular patterns via PRRs such as TLRs [25]. During the inflammatory state, TLRs interact with pathogens and their molecules such as LPS, flagella or DNA, resulting in activation of IECs that affects their cellular functions including production of cytokine/chemokines and other factors [24]. Some studies have evaluated the effects of *Lactobacillus* on inhibition of *Salmonella* infection in IECs [18, 19]; however, the molecular mechanisms underlying these effects are not yet completely understood. Therefore, we employed an *in vitro* epithelial cell culture system to investigate the immunoregulatory activity of LAB strains that were isolated from different sources of Korean foods. The results revealed that LABs could interact with IECs and reveal strain-specific probiotic effects. Therefore, we examined the anti-inflammatory activities of three different LAB strains against *Salmonella* inflammatory response in HT-29 cells. Cytokines such as IL-6, IL-8, MCP-1 and IL-1β are potent pro-inflammatory cytokines, and their elevated levels play crucial roles in the development of several diseases including diarrhea and inflammatory bowel diseases. It has also been reported that *Salmonella* infection increased the levels of IL-6, IL-8, and IL-1β in the colons of pigs [28]. Moreover, exposure of IECs to *Salmonella* induced the production of IL-6, IL-8, and MCP-1 *in vitro* [29, 30]. The results of the present study showed that HT-29 cells treated with *Salmonella* increased the expression of IL-6, IL-8, MCP-1 and IL-1β, but that these increases were differentially altered when cells were pretreated with LAB strains. All LABs, especially *L*. *brevis*, remarkably diminished the mRNA levels of all four pro-inflammatory cytokines in HT-29 cells that were post-treated with *Salmonella* for 12 h. Similarly, *L*. *paracasei* CNCMI-4034 was able to attenuate inflammation induced by *Salmonella* in human dendritic cells (DCs), while it significantly decreased the production of IL-6, IL-8 and MCP-1 in DCs [31]. Another *in vitro* study showed that *Lactobacillus* pre-treatment decreased the level of IL-8 in Caco2 cells that had been stimulated with *Salmonella* [18]. In addition, the expression of IL-1β in *L*. *acidophilus* treated rats was significantly lower than that of *Salmonella* alone [32]. Oral administration of *L*. *casei* abrogated *Salmonella* induced IL-6 and IL-1β expression in mice [33]. Taken together, the results of these studies support our finding that *Lactobacillus* strains have the potential to inhibit inflammatory cytokine production induced by *Salmonella in vitro* in IECs.

TNF-α is a prominent pro-inflammatory cytokine associated with inflammatory state and intestinal diseases [34]. In the chronic inflammatory state, host immune responses were dysfunctional or weakened by TNF-α through the suppression of receptor expression or transduction. It has also been reported that elevated level of TNF-α reduce protection against microbes and induce lengthening of inflammation via dysregulation of immune inflammatory responses [35]. HBD-2 is a cysteine-rich inducible antimicrobial protein that plays a crucial role in host defense mechanisms against pathogenic bacteria and viruses [36]. The presence of TNF-α induced production of human β-defensin-2 (HBD-2) in human intestinal (Caco2) and lung ECs [36, 37]. We also found similar results in our study. Treatment of Caco2 cells with TNF-α increased the expression of HBD-2 *in vitro*; however, Caco2 cells treated with LABs differentially regulated HBD-2 expression *in vitro*. *L*. *brevis* further increased the HBD-2 level in Caco2 cells post-treated with TNF-α. Similarly, Schlee et al. [38] found that probiotic *E*. *coli* Nissle 1917 was able to induce HBD-2 production in Caco2 cells. The induction of HBD-2 production by probiotics mainly occurred through NF-κB and AP-1 dependent pathways.

TLRs are the primary sensors of pathogenic invaders and their prolonged or over activation induces adverse effects on the host. Several negative regulators target TLRs signaling and control their excess activation directly or indirectly [39]. Upon TLRs interaction with ligands, MyD88 or TRIF recruit an array of negative regulatory proteins such as A20, SIGIRR, Tollip, IRAKM, and Bcl3 to activate transcription factors including c-JUN, NF-κB and ATF-2, which positively regulate transcription of genes related to inflammatory signaling. A20 is the first deubiquitination enzyme that rapidly induces or inhibits TNF-mediated NF-κB activation. Following TLR ligation, subsequent activation of the NF-κB was tightly controlled by A20 via removal of K63-linked polyubiquitin chains from TNFR-associated factor 6 (TRAF6) [40]. Additionally, porcine IECs (PIE) treated with *L*. *jensenii* TL2937 and *Bifidobacteria* up-regulated the expression of A20 relative to the control [23, 41]. The results of the present study also showed that pre-stimulation with LABs increased the A20 level in HT-29 cells that were post-stimulated with *Salmonella*. In addition, LABs stimulation was capable of increasing the levels of toll interacting protein (Tollip) and single immunoglobulin IL-1 related receptor (SIGIRR) in HT-29 cells. SIGIRR is a negative regulator of IL-1 and TLR signaling, and its deficiency could make mice more susceptible to enterohemorrhagic *E*. *coli* and *Salmonella* [42]. Tomosada et al. [41] reported that LABs pre-stimulation increased the mRNA level of Tollip and SIGIRR in PIE cells treated with enterotoxigenic *E*. *coli*. In addition, IRAKM belongs to the IRAK family. Upon TLRs stimulation, IRAKM is activated and negatively regulates TLR signaling by preventing the dissociation of IRAK and IRAK-4 from MyD88 and IRAK-TRAF6 complex formation [43]. The absence of IRAKM in mice and macrophages resulted in increased production of cytokines in response to TLR/IL-1 stimulation and bacterial challenge. Moreover, phosphorylation of p38, JNK, and ERK MAPKs and the degradation of IκB-α were found to be higher in the IRAKM deficient macrophages [43]. Our results showed that *L*. *brevis* stimulation increased the mRNA level of IRAKM in HT-29 cells treated with *Salmonella*. However, other LAB strains did not show higher levels of IRAKM that were relatively similar to *Salmonella* alone, suggesting that isolated LABs were beneficially modulated negative regulators of TLR signaling in HT-29.

The intestinal epithelial barrier is a form of complex system that reduces diffusion or permeability of microbes and their cellular components across the intestinal epithelium. The paracellular spaces between the IECs are connected each other by Tight-Junction (TJ) proteins including ZO-1 and occludin. External stimuli such as enteric pathogens are capable of disturbing TJ and increasing paracellular permeability via different mechanisms [44]. For example, infection by *Salmonella* and its effector proteins increases intestinal permeability via perijunctional contractions or activation of geranylated proteins [45, 46]. Another *in vitro* study reported that *S*. *typhimurium* infection disrupted the intestinal barrier via dephosphorylation of occludin and ZO-1 proteins in T84 IECs [47]. We found similar results when HT-29 cells were treated with *Salmonella*. It has been reported that probiotics could improve intestinal barrier integrity via JNK and other pathways, while treatment with *L*. *plantarum* has been shown to protect the intestinal barrier function of porcine IE J2 cells (IPEC-J2) and weaned piglets infected with enterotoxigenic *E*. *coli* by increasing the expression of occludin and ZO-1 proteins [48, 49]. Similarly, HT-29 cells pre-treated with LABs alone or in combination with *Salmonella* or post-treatment with *Salmonella* increased the ZO-1 level compared with *Salmonella* alone, suggesting that LABs could protect TJ against *Salmonella*.

*Salmonella* infection has also been shown to increase the expression of TNF-α, which is a potent pleiotropic inflammatory cytokine, *in vitro* and *in vivo*; therefore, it is often associated with many diseases including inflammation [32, 50]. Similarly, HT-29 cells treated with *Salmonella* increased the production of TNF-α in HT-29 cells, as well as PBMC cells. Conversely, LAB treatment diminished *Salmonella* induced TNF-α production in HT-29 cells. Treatment with all LABs also indirectly suppressed PBMC cells to produce lower level of TNF-α. These results agree well with those of Huang et al. [50] who reported that *L*. *acidophilus* was able to reduce TNF-α production in Caco2 cells infected with *Salmonella*. In addition, lower level of TNF-α was observed in *L*. *paracasei* and its cell free supernatant (CFS) treated human DCs via TLR activation [31]. We also found that LABs stimulation increased the expression of IL-10 and TGF-β against *Salmonella* in HT-29 cells. IL-10 is a key immunoregulatory cytokine that plays a crucial role in inhibition of inflammatory diseases, and its modulation by probiotic administration has been observed in ulcerative and IBD patients [51]. Similarly, TGF-β is a potent immunoregulatory cytokine involved in increasing the production of IgA [52]. Therefore, higher levels of IL-10 and TGF-β and lower level of TNF-α indicate the anti-inflammatory activity of our isolated *Lactobacillus* strains in HT-29 cells, demonstrating that LABs restore homeostasis and inhibit *Salmonella* induced inflammatory response in HT-29 cells.

Nuclear factor-kappa B (NF-κB) is a central regulator of inflammatory signaling induced by enteric or enteroinvasive pathogens *in vitro* or *in vivo* [53]. Infection by *Salmonella* results in activation of NF-κB in intestinal ECs and leads to up-regulation of the transcription of genes related to inflammatory cytokines/chemokines [54]. In the present study, we also found that *Salmonella* treatment increased phosphorylation of IκB-α in HT-29 cells. Post-stimulation with LABs inhibited *Salmonella* induced NF-κB activation by reducing phosphorylation of IκB-α in HT-29 cells, which is similar to the results of other studies for *L*. *acidophilus* [50], *Bifidobacterium infantis* and *L*. *salivarius* [55].

In conclusion, the results of the present study demonstrate that *Lactobacillus* strains isolated from Korean foods are beneficial for attenuation of inflammatory responses induced by *Salmonella in vitro*. HT-29 cells differentially responded to LABs and *Salmonella* by producing various levels of cytokines/chemokines. Additionally, *Lactobacillus* strains were able to improve innate immune responses against *Salmonella* by decreasing the production of different inflammatory cytokines and modulating expression of negative regulators of TLRs and activation of the NF-κB pathway. Overall, this study increases our understanding of the molecular mechanisms involved in the immunoregulatory activity of probiotics against enteric pathogens.
